# A Review of Emerging Immunotherapeutic Strategies for IDH-Mutant Glioma

**DOI:** 10.3390/cancers17132178

**Published:** 2025-06-27

**Authors:** Masih Tazhibi, Eric P. Grewal, Rishab Ramapriyan, Leland G. K. Richardson, Gust Vandecandelaere, Adrian Kalaw, Parker Kotlarz, Samuel J. Steuart, Jing Sun, Matthew Gaffey, Daniel P. Cahill, Julie J. Miller, William T. Curry, Bryan D. Choi

**Affiliations:** 1 Brain Tumor Immunotherapy Laboratory, Massachusetts General Hospital, Boston, MA 02114, USA; 2 Department of Neurosurgery, Mass General Brigham, Boston, MA 02114, USA; 3 Harvard Medical School, Boston, MA 02115, USA; 4 Department of Pathology, Mass General Brigham, Boston, MA 02114, USA; 5 Faculty of Medicine, Katholieke Universiteit Leuven, 3000 Leuven, Belgium; 6 Pappas Center for Neuro-Oncology, Department of Neurology, Mass General Brigham, Boston, MA 02114, USA

**Keywords:** glioma, isocitrate dehydrogenase, neurosurgery, immunotherapy, tumor microenvironment, tumor antigens

## Abstract

IDH-mutant gliomas (IMGs) are a unique subset of diffuse gliomas that follow a relatively indolent course compared to IDH-wildtype glioblastoma (GBM) but inevitably progress, often to a higher histologic grade. Current standard therapies, including surgery, chemoradiation, and the recently approved mutant IDH inhibitor (mIDHi) vorasidenib, provide limited disease control and are not curative. Given the immunosuppressive tumor microenvironment (TME) driven by the mutant IDH enzyme and its associated oncometabolite 2-hydroxyglutarate (2-HG), novel immunotherapies offer a promising avenue for treatment. The goal of this paper is to review the main immunologic characteristics that distinguish IMG from GBM, including reduced T cell infiltration and function, fewer myeloid cells, and increased immune-dampening signaling. We also evaluate the preclinical and clinical evidence for immunotherapeutic approaches with the most potential to induce meaningful clinical activity, such as immune checkpoint inhibitors, CAR T cells, tumor vaccines, myeloid redirection, and oncolytic viruses. Despite significant advances in immunotherapy for IMG, fundamental questions persist, including optimal timing and combination strategies, mechanisms underpinning treatment resistance, and strategies to overcome the suppressive microenvironment. Future exploration of these treatment modalities, with a focus on mitigating soluble immunosuppressive factors in the TME, enhancing in situ T cell persistence, and leveraging novel antigen targets, is critical for advancing the state of therapy for this presently incurable group of tumors.

## Simple Summary

IDH-mutant gliomas are a type of brain tumor that grow more slowly than IDH-wildtype glioblastomas but eventually become more aggressive. Current treatments, such as surgery, chemotherapy, radiation, and the new drug vorasidenib, help manage the disease but are not curative. Because these tumors create an environment that weakens immune responses, researchers are exploring new treatments that use the body’s own defenses. Unfortunately, standard immunotherapies like immune checkpoint inhibitors have not worked well for gliomas on their own. However, combining these drugs with other approaches may improve outcomes. Furthermore, several other treatment avenues are also being developed, including vaccines that teach the immune system to recognize the cancer, engineered immune cells that can directly attack the tumor, and viruses that selectively infect and destroy cancer cells. While several challenges remain, immunotherapy holds significant promise in improving outcomes for patients with this devastating disease.

## 1. Introduction

Diffuse gliomas are the largest group of malignant brain tumors in adults, imparting near-universal fatality despite standard therapies [1]. Unique among these tumors is isocitrate dehydrogenase (IDH)-mutant glioma, a subset of malignancies that predominates earlier in adulthood, demonstrates slower growth, and often displays long intervals of stable disease between progression events [2]. IDH-mutant glioma (IMG) includes two distinctive molecularly and morphologically defined entities (astrocytoma or oligodendroglioma), and can present as a low-grade (grade 2) or high-grade (grade 3 or 4) tumor.

Despite a comparatively indolent course relative to IDH-wildtype gliomas such as glioblastoma (GBM), IMGs inevitably recur, often having transformed to a higher grade [3]. Current standard-of-care therapies include maximal safe resection and adjuvant chemoradiation (CRT) with temozolomide or combination procarbazine, lomustine, and vincristine (PCV), which potentiate limited disease control and often result in long-term toxicities [4,5]. Recently, the mutant IDH inhibitor (mIDHi) vorasidenib was approved for treatment of low-grade tumors, marking a significant advance in therapeutics development, but even this medication provides a moderate progression-free survival benefit and is not curative. Thus, novel therapeutic approaches are needed [6].

Over the last decade, a growing number of studies have shown IMG to possess unique biological and immunologic properties that present a potential opportunity for cancer immunotherapy [7]. It is now apparent that the IDH mutation confers an altered epigenetic, antigenic, and metabolic profile within tumors that may enhance susceptibility to targeted or immune-based therapies [5,8]. With multiple modalities of immunotherapy now readily deployable—such as immune checkpoint inhibitor antibodies, tumor-specific antigen vaccines, and chimeric antigen receptor (CAR) T cells—such approaches may also be adapted to delay or prevent future malignant transformation of IMG.

As such, there is a compelling case for continued exploration and development of immunotherapies in IMG. The goal of this review is to discuss the distinct immunologic characteristics of IMGs, lessons learned to date with existing experimental immunotherapies, and emerging therapeutics and future directions.

## 2. IDH-Mutant Glioma (IMG)

### 2.1. Immune Landscape of IMG

The glioma tumor microenvironment (TME) comprises complex interactions between tumor cells, non-cancerous brain stroma, and recruited elements such as immune cells, fibroblasts, endothelial cells, and extracellular matrix [9]. The immune composition of gliomas significantly impacts tumor progression and response to immunotherapy [10]. Classically, gliomas have been described as immunologically silent compared to other cancers [11]. This is at least somewhat attributable to IDH mutations, which confer a “colder” immune phenotype in IDH-mutant compared to wildtype tumors, though this paradigm is an oversimplification [12].

Over the past decade, advances in immune profiling technologies have enabled us to go beyond “coldness” and document key biological factors that specifically define the TME of IMGs relative to GBM (Table 1). Quantitatively, IMGs are infiltrated by fewer lymphocytes, including both CD4+ helper T cells and CD8+ cytotoxic lymphocytes [13,14]. Reductions in regulatory T cells (Tregs), which are abundant within GBM and correlate negatively with survival, have also been reported in IDH-mutant tumors compared to wildtype controls [15,16]. Of note, this may be attributable to decreases in chemokines such as CCL2, a factor often enriched in GBM that has been shown to attract Tregs and MDSC precursors from the circulation [17].

It is well established that the function and phenotype of infiltrating immune cells are also shaped by IDH mutational status. IMGs exhibit less tumor-infiltrating lymphocyte (TIL) infiltration and PD-L1 expression compared to IDH-wildtype gliomas, with reduced PD-L1 expression associated with increased gene promoter methylation [14]. Single-cell analyses report lower levels of cytotoxicity and interferon-related immune signatures among CD8+ and CD4+ T cells in IDH-mutant tumors compared to the wildtype phenotype [18,19]. IMGs also harbor a greater number of tumor-resident microglia, contrasting with wildtype tumors, which are more infiltrated by monocyte-derived macrophages [20].

Recently, our group reported on the effects of mutant IDH on the intratumoral myeloid compartment associated with gliomas [21]. We performed RNA sequencing and enumerated myeloid cells within newly diagnosed, treatment-naive specimens of human IDH-mutant grade 4 astrocytoma and IDH-wildtype glioblastoma (GBM) and developed a syngeneic mouse model to compare glioma lines differing only in IDH mutation status. We found that IDH-mutant specimens contained fewer intratumoral M2-like macrophages and myeloid-derived suppressor cells (MDSCs) than their wildtype counterparts, and that this phenomenon could be reproduced in mice by introducing the IDH-mutant enzyme into murine glioma models.

Soluble mediators are thought to play a key role in shaping the glioma TME. IDH-mutant tumors are known to be influenced by 2-hydroxyglutarate (2-HG), an oncometabolite produced by the mutant IDH enzyme [12,22,23,24]. 2-HG leads to widespread glioma intrinsic reprogramming by inhibiting α-ketoglutarate-dependent histone demethylase enzymes [22]. As previously described, this has several direct and indirect effects on the TME [7]. Directly, 2-HG significantly impairs T cell proliferation and antigen-specific responses by inhibiting ATP-dependent T cell receptor (TCR) signaling events, such as Fyn and phospholipase C-γ1 (PLC-γ1) phosphorylation [25]. 2-HG also dampens calcium influx in stimulated T cells, significantly reducing nuclear factor of activated T cells (NFAT) activity and expression of factors related to activation and effector function (i.e., IFN-γ, IL-2, and PD-1) [25]. 2-HG also leads to metabolic derangement in T cells by inhibiting mitochondrial ATP production, activating AMPK, and disrupting polyamine biosynthesis [25]. Beyond T cells, 2-HG also alters the phenotype of tumor-associated macrophages (TAMs), producing an immunosuppressive environment by altering tryptophan metabolism and aryl hydrocarbon receptor (AhR) activation [26]. These changes lead to increased immunosuppressive cytokines and decreased expression of co-stimulatory molecules and antigen presentation machinery. Indirectly, 2-HG interferes with CD8+ T cell recruitment to the tumor site, potentially by suppressing interferon-γ-inducible chemokines (i.e., CXCL10) [13]. 2-HG also downregulates ULBP1 and ULBP3 on tumor cells, promoting escape from natural killer cells [27].

Another key soluble mediator of immunosuppression within gliomas is VEGFA. Compared to lower-grade tumors, higher-grade IMGs are associated with increased VEGFA signaling as well as decreased expression of L-selectin, a molecule involved in lymphocyte infiltration and memory T cell function [21]. In addition to its angiogenic properties, VEGFA is a documented immunosuppressant, activating Tregs, recruiting myeloid derived suppressor cells (MDSCs), polarizing TAMs, inhibiting dendritic cell maturation, and promoting CD8+ T cell exhaustion, all of which reduce effector T cell activation and proliferation [28]. Though effective in reducing tumor-associated edema, clinical trials to date have failed to achieve significant benefit with the anti-VEGF agent bevacizumab in the context of immunotherapy [29,30,31,32].

### 2.2. Current Therapeutic Approaches for IMG

#### 2.2.1. Immune Checkpoint Inhibitors

**Figure 1:**
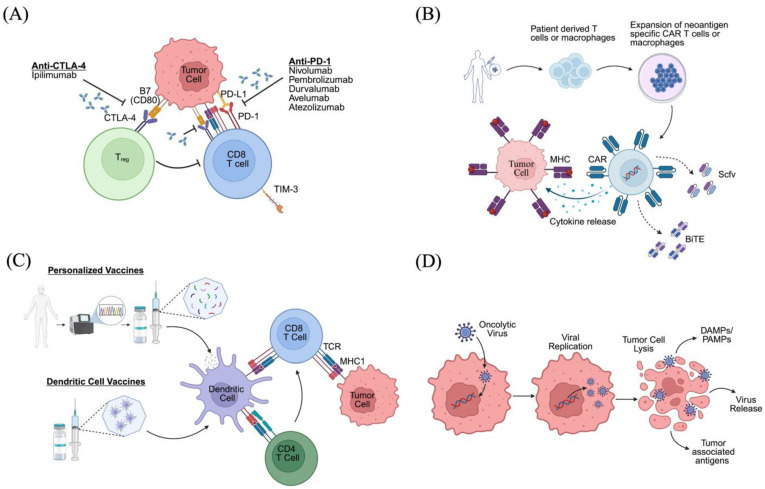
Current major immunotherapeutic strategies for glioma. (**A**) Immune checkpoint inhibitors, (**B**) CAR T cells and macrophages, (**C**) tumor vaccines, (**D**) oncolytic viruses. Created with BioRender.com. Tazhibi, M. (2025) https://BioRender.com/h42n006 (accessed on 5 March 2025).

Immune checkpoint inhibitors (ICIs) are a powerful class of antibody-based immunotherapies that can reinvigorate tumor-specific T cells by blocking inhibitory ligands such as PD-1/PD-L1, CTLA-4, and LAG-3 (Figure 1A). Despite remarkable efficacy in metastatic melanoma (including cases with brain metastases), non-small cell lung carcinoma, renal cell carcinoma, and other tumors, no ICI monotherapy to date has demonstrated efficacy in primary brain tumors [33,34,35]. For example, the results of the highly anticipated phase III CheckMate 143 study, which was the first major randomized clinical trial assessing the inhibition of the programmed death pathway for the treatment of recurrent GBM, did not show an improvement in overall survival when comparing nivolumab to bevacizumab [36]. One possible insight into this failure may be reflected by a unique population of PD-1^hi^ Tregs identified within both the tumor and peripheral blood of study participants, including subjects with IMG [37]. After nivolumab infusion, these cells were shown to produce increased IFN-γ when restimulated in vitro. However, this effect was accompanied by an even greater increase in exhaustion and T cell dysfunction, suggesting that T cell reprogramming may not be as readily achieved as in other tumor contexts where ICIs have succeeded. Another potential explanation for the failure of CheckMate 143 is inefficient delivery of nivolumab across the blood–brain barrier as a result of its relatively large molecular size [38]. In recurrent glioblastoma, most antigen-specific T cells are already sequestered at the tumor site, suggesting that a substantial population of dysfunctional T cells within the tumor microenvironment may have been essentially untouched by PD-1 blockade [38]. Nevertheless, several ICIs remain under investigation for IMG, both as monotherapy and in combination regimens (Table 2) [39].

As a monotherapy, ICIs face multiple serious biological challenges unique to IMG. For one, 2-HG enhances DNA methylation and PD-L1 suppression in diffuse gliomas, potentially limiting target expression [14,40]. As reviewed above, IMGs also harbor fewer intrinsic lymphocytes that may be tumor-specific, limiting the repertoire of tumor-targeting T cells that could be released by ICIs.

However, it is generally appreciated that ICIs might be optimally deployed alongside other agents that target additional arms of tumor immune suppression [41,42]. One such agent is vorasidenib, the first mIDHi approved for IDH-mutant low-grade gliomas. In theory, reversal of 2-HG-mediated immune suppression by mIDHi may lower the threshold for successful ICI activity in a combination approach [21]. This has been explored preclinically, where the addition of PD-L1 blockade to mIDHi showed significant therapeutic advantages in an IDH-mutant mouse glioma model by promoting the generation of memory CD8+ T cells and mitigating T cell exhaustion [43]. Of note, a phase I trial evaluating the combination of vorasidenib and pembrolizumab for the treatment of recurrent or progressive IDH1-mutant glioma is currently underway (NCT05484622). Furthermore, a phase II trial by the University of Pittsburgh combining nivolumab and an alternate mIDHi, ivosidenib, was completed in 2023, with results recently posted (NCT04056910). Notably, 85.7% of participants analyzed in the combination group had stable disease eight weeks following initial treatment. Six-month progression-free survival was 20% and no dose-limiting toxicities were reported.

Beyond IDH inhibition, ICIs are also being tested in combination with tumor vaccines. One notable example is a phase I clinical trial from the German Cancer Research Center that employs a combinatorial treatment of IDH1-R132H-specific vaccination with the PD-L1 inhibitor avelumab (NCT04056910).

#### 2.2.2. CAR T Cell Therapy

CAR T cells are synthetically engineered T lymphocytes that can be directed against virtually any surface antigen of choice (Figure 1B). First-generation CAR T cells have an extracellular antigen recognition domain, a transmembrane domain, and an intracellular signaling domain. Second-generation CAR T cells include an additional co-stimulatory domain to enhance activation and persistence [44]. Third-generation CAR T cells have multiple co-stimulatory domains for greater efficacy [45]. More advanced CAR T cells, such as those secreting bispecific T cell engagers (BiTEs), T cell-engaging antibody molecules (TEAMs), or other bioactive molecules, are specifically designed to recruit and activate other immune cells or otherwise modulate the TME [46,47].

CAR T cell therapy has demonstrated remarkable efficacy in B cell malignancies such as non-Hodgkin lymphoma, acute lymphoblastic leukemia, and multiple myeloma [48,49]. However, CAR T cell therapy for brain tumors, including glioma, has faced several limitations. These include extensive intratumoral heterogeneity promoting antigen escape and treatment resistance, unique anatomical features like the blood–brain barrier (BBB) which restrict T cell infiltration into the CNS, and T cell exhaustion via upregulated metabolic competition, intratumoral hypoxia, release of inhibitory cytokines, and suppressive myeloid cells [50]. Nonetheless, multiple phase I clinical trials of CAR T cell products have been deployed in glioma, with a specific focus on GBM-specific antigens such as EGFR and EGFRvIII, IL-13Rα2, and B7-H3 [51,52]. Among these, a recent study that tested an IL-13Rα2-specific CAR T cell product in GBM did not exclude IMGs within the treatment cohort [53]. Among the 54 enrollees evaluable for response, all 3 subjects who demonstrated objective responses had grade 3 IMGs. While no other major clinical trials have included large cohorts of patients with IMGs, these potential signals suggest that future trials should continue to include these patients within treatment arms. Additional approaches utilized in GBM may also be applied to IMG. For instance, EGFRvIII, while primarily associated with the wildtype phenotype, has been observed in some IMGs as well and may be targetable for a subset of patients [43,54]. Furthermore, platelet-derived growth factor receptor alpha (PDGFRA), a receptor tyrosine kinase amplified in IMGs and linked with poor prognosis, has been effectively targeted by CAR T cells in other cancers like rhabdomyosarcoma [55,56].

Several challenges remain for the effective translation of CAR T therapy for IMGs, such as a need to better understand and overcome the hostile TME and achieve improved T cell penetration into the tumor site [57]. Among these challenges is the potential for antigen escape, arising from extensive heterogeneity in gliomas making it difficult for CAR T cells targeting a single antigen to achieve comprehensive tumor control [58]. This phenomenon has been well documented in GBM clinical trials of CAR T cells and may be plausibly encountered with IMGs as well, particularly in high-grade tumors [51]. CAR T cell exhaustion, resulting from chronic antigen exposure, persistent immunosuppressive signaling, and upregulated PD-1/LAG-3 expression, is another critical limitation to contend with [58]. Clinical and preclinical evidence suggests that lymphodepletion may be a requirement for effective CAR T cell expansion and endurance [59,60,61]. Finally, combination approaches and optimization of administration techniques (i.e., intravenous or locoregional delivery, hydrogels, focused ultrasound, etc.) may address the technical challenges of delivering CAR T cells across the blood–brain barrier and into the tumor site [62,63,64].

#### 2.2.3. Myeloid Redirection and CAR Macrophages

One of the most striking differences between IMG and GBM with respect to the myeloid compartment is the downregulation of leukocyte chemotaxis and decreased infiltration of M2-like macrophages and MDSCs in the IMG TME [17,65]. Given the high number of TAMs in GBM and their roles in promoting tumor progression through facilitating angiogenesis, tumor invasion and metastasis, and TME modification, TAMs are now being targeted through experimental therapies aimed at reduction and repolarization [65,66,67]. A variety of strategies have been developed for redirecting myeloid cells to target GBM, but little comparable work has been conducted in the context of IMGs.

One promising myeloid-based therapy for GBM is CAR macrophage therapy (Figure 1B). This approach uses similar principles to CAR T cell therapy but leverages the capacity of macrophages for phagocytosis, antigen presentation, and tumor infiltration. Like CAR T cells, CAR macrophages can either be generated in vitro and injected intracranially or generated in vivo through injection of nanoparticle-formulated nucleic acids or engineered viral vectors. EGFRvIII/GPC3-targeting CARs have been induced in pluripotent stem cell-derived macrophages that demonstrated improved antitumor effect through M1 polarization, tumor cell phagocytosis, and apoptotic tumor cell efferocytosis [68]. In vivo CAR macrophages targeting CD133 on glioma stem cells and HER2 on brain stem gliomas have also been demonstrated in mouse tumor models and patient-derived xenograft models [69,70]. Notably, one benefit of in vivo generation of CAR macrophages is that this strategy may allow for the reprograming of M2-like TAMs to an antitumor M1-like phenotype. There are currently three clinical trials using CAR macrophages for the treatment of malignancy (NCT04660929, NCT05007379, NCT06224738) but none are focused on brain tumors. Despite the need for additional work, these preliminary results highlight the promise of this rapidly developing platform.

#### 2.2.4. Tumor Vaccines

Tumor antigen-specific vaccines are a promising approach for treating IMGs (Figure 1C). IDH mutations, particularly IDH1-R132H, create a homogenous and highly expressed tumor-specific neoantigen which elicits T_H_1 polarization and production of mutation-specific antibodies [42,71,72].

Preclinical research has demonstrated the potential effectiveness of IDH1-R132H peptide vaccines. In MHC-humanized mice, the administration of the IDH1-R132H P123-142 peptide vaccine elicited therapeutic helper T cell responses and mutation-specific antibodies. Intracranial glioma models with the IDH1-R132H mutation have also been developed [73]. In these mice, treatment with an mIDH1 peptide led to increased peripheral CD8+ T cells, IFN-γ production, and anti-IDH1 mutant antibodies, decreased TGF-β2 and IL-10, and significantly lengthened survival.

Preclinical studies have also explored combination therapies and alternative vaccine strategies. The use of synthetic peptides created from glioma-associated antigens significantly prolonged survival in vaccinated mice with IDH-mutant tumors when treated with an IDH inhibitor [13]. Furthermore, in a murine IMG model, tumor growth was significantly reduced following adoptive transfer of T cells from mice vaccinated with an IDH1-R132H peptide vaccine, an effect only observed in combination with an IDH inhibitor [25].

These promising preclinical findings led to clinical trials investigating IDH1-R132H peptide vaccines in patients with IMGs. The NOA-16 trial, a first-in-human, multicenter, phase I study combining vaccination with radiotherapy and adjuvant temozolomide, reported encouraging early results [74]. In total, 93.3% of enrolled patients achieved vaccine-induced immunity, and the three-year survival rate was 84%. A significant positive correlation was observed between tumoral IDH1-R132H peptide presentation and peripheral T cell responses. Furthermore, “pseudoprogression” following vaccination correlated with increased penetration of IDH-mutant-specific T cells, potentially representing intratumoral inflammation.

The results of several other studies are highly anticipated, including those of the ongoing trial of an IDH1-R132H dendritic cell vaccine in patients with IMG (NCT02771301), the recently completed RESIST trial which investigated the use of PEPIDH1M for patients with recurrent grade 2 gliomas (NCT02193347), and the newly opened ViCToRy trial combining PEPIDH1M with the dual IDH1/2 inhibitor vorasidenib (NCT05609994).

While tumor vaccines are a facile means of inducing tumor immunity, antigen-specific T cells must still traffic into the tumor and overcome TME-mediated immune suppression. Particularly given the unique, 2-HG-rich TME of IMG, effective vaccine strategies may be particularly dependent on adjunctive agents such as mIDHi or VEGF blockade to unlock effective T cell killing.

#### 2.2.5. Oncolytic Viruses

OVs are genetically modified or naturally existing viruses that selectively reproduce and lyse in tumor cells while sparing healthy cells (Figure 1D) [54,75]. These preferential tumor-lysing viruses can additionally modify the TME and deliver therapeutic dosing to the local area [76,77,78]. OVs are often combined with co-therapies to boost their effect and can be delivered systemically or intratumorally [79,80]. The first and only approved OV by the FDA is Talimogene laherparepvec (T-VEC), an engineered herpes simplex virus type 1 (HSV1) to treat metastatic melanoma. [81]. Other OVs that have been approved abroad include H101 (nasopharyngeal carcinoma, China) and ECHO-7 (Stage I–II melanoma, Armenia, Georgia, Latvia, discontinued due to manufacturing issues in 2019) [82]. More recently, Teserpaturev (G47Δ), a triple-mutated HSV1 virus, was approved in Japan for glioblastoma following radiotherapy and temozolomide [83,84]. Other genetically engineered OVs are also currently undergoing investigation for GBM (NCT02062827, NCT05717712).

Recent work has also begun exploring the treatment effects of OVs in IMG. Notably, in G47Δ, a post hoc analysis showed that IDH1 mutation status did not affect median overall survival from the time of initial diagnosis/surgery or Teserpaturev initiation [83]. Similarly, a phase I trial of a measles-based OV (MV-CEA) in glioma showed no significant difference in median PFS by IDH status, though interpretation was limited by the low number of IDH-mutant cases (*n* = 2/22) [85]. Notably, an HSV-based OV (CAN-3110) has shown longer survival for HSV1-seropositive patients, though analysis focused on IDH-wildtype gliomas due to few IDH-mutant cases (*n* = 4/41) [86]. A key question from these studies is whether OV therapy can enhance endogenous T cell responses to IMG, which has not been demonstrated in vitro to date. Furthermore, an open question is whether OV-induced inflammation may overcome 2-HG-mediated suppression, a large feat given the near-uniform expression of mIDH across IMG cells. More research needs to be conducted on OVs deployed specifically in IMG patients.

### 2.3. Future Directions for Immunotherapy in IMG

The panoply of immunotherapeutic approaches currently being evaluated preclinically and clinically for IMG reflects the many potential avenues for inducing antitumor immunity. However, a successful immunotherapy for IMG hinges on overcoming the unique challenges posed by its unique TME and fully harnessing therapeutic vulnerabilities.

#### 2.3.1. Optimizing Modulation of the TME

A major direction in future research will be to develop therapies that successfully modulate and reprogram the IDH-mutant TME, which is heavily shaped by 2-HG. This is particularly important in the era of mIDHi, which might not only affect tumor cells intrinsically, but may also affect tumor-infiltrating lymphocytes and myeloid cells. While the inhibitory effect of 2-HG on T cells has been well characterized, a mounting body of evidence suggests 2-HG might play a role in counteracting suppressive myeloid cell populations [65,87]. Thus, strategies that employ cell-specific mIDHi, or regimens that counteract this potentially deleterious myeloid-specific effect with myeloid repolarization strategies, should be employed.

An additional immunosuppressive factor to contend with in IMG is VEGFA. Recently, our group demonstrated that upon progression to a higher grade, recurrent IDH-mutant astrocytomas uniquely upregulate VEGFA, which also correlates with counterproductive alterations in the TME [21]. Strategies that block VEGFA, particularly in combination with therapies that aim to boost endogenous T cell responses or employ CAR T cells, should be considered to produce a more hospitable TME that promotes and maintains optimal activation of cellular effectors [88].

It will also be important to determine how standard treatments like temozolomide influence the TME to better optimize combination therapies. While alkylating agents like temozolomide may induce a higher tumor mutational burden and enhance immune recognition, combined treatment with RT and temozolomide has also been shown to concomitantly increase VEGFR1 expression and Treg infiltration, with prolonged treatment (≥10 courses of TMZ) exhibiting a further increase in Foxp3+ Tregs as well as a decrease in CD163+ cells. While it is unclear whether similar effects occur in IMG, the Stupp protocol may produce certain immunosuppressive alterations in the glioma microenvironment that require further evaluation [89]. Conversely, emerging evidence suggests that treatments like temozolomide can also drive glioma cell senescence, in both GBM and IMG models [90]. In these studies, senescence was characterized by DNA damage, which could have the effect of inducing inflammation through the cGAS-STING pathway, as well as producing nonsynonymous mutations that may serve as neoantigens [91]. In other contexts, senescence can also lead to the expression of extracellular antigens such as uPAR, which may be amenable to antibody or CAR T-based therapy [92]. Though not reconciled in patient investigations to date, this dichotomy of counterproductive TME changes and favorable tumor-intrinsic alterations warrants future study.

Ultimately, while many of the insights and approaches discovered thus far in IMG are supported by promising preclinical data, differences in human tumor biology and immune system may impact the efficacy and relevance of strategies developed in animal models. These factors highlight the need for care when interpreting preclinical findings and the importance of developing and validating more representative models better capturing the unique biology of human IMG.

#### 2.3.2. Enhancing Antigen-Specific T Cell Responses and Exploring Novel Antigenic Targets

A major therapeutic hurdle in IMGs is the limited presence of intratumoral T cells. While mIDHi may provide a means of augmenting T cell persistence within the TME, antigen-specific approaches like IDH1-R132H neoantigen vaccination are particularly promising for boosting endogenous immunity. Clinical experience with neoantigen vaccines in solid tumors has revealed not only evidence of inducing antigen-specific immune responses in patients, but also the possibility of epitope spreading, which releases additional neoantigens upon induction of successful tumor lysis [93]. Perhaps in combination with mIDHi and additional T cell modulators such as ICIs, tumor vaccines may serve as a means of boosting T cell trafficking into tumors to recognize shared as well as patient-specific targets.

Even in tumors harboring sufficient TILs, it has been documented that T cells preferentially localize in the perivascular zones within IMG, limiting deep tumor penetration and potentially contributing to treatment resistance [94,95]. While BBB penetration is likely not a limiting factor in these cases, this highlights the need for future work characterizing mechanisms of intratumoral T cell exclusion, as well as strategies such as chemokine modulation which may enable deeper penetration of TILs.

Defining new glioma antigens should also be a major priority for future research. Emerging markers specific to IMG are promising targets for synthetic immunotherapy approaches like CAR T cell therapy and BiTEs. Beyond overexpressed markers, however, recent advances in cellular immunotherapy have also made it possible to target tumor-specific antigens in the context of MHC, as demonstrated using TCR-T cells in preclinical GBM models [96,97]. Such an approach has the potential to bypass endogenous immunity and directly deliver antigen-specific T cells to the tumor site, and MHC-bound antigens unique to IMGs should continue to be mined.

#### 2.3.3. Patient Selection, Stratification, and Immune Biomarker Selection

Though IMGs share many similarities as a group, they consist of five unique malignancies when divided by entity and grade. Previous work from our group and others has shown that each type of IMG has characteristic alterations in tumor immunologic status that may differentially affect the outcome of immunotherapy [21,98]. Notably, the large-scale INDIGO trial of vorasidenib only focused on grade 2 IDH-mutant oligodendroglioma and astrocytoma. Thus, it remains important to define at which point experimental immunotherapies are optimally deployed in patients with IMG, who may present throughout the course of their disease with tumors at different stages. Given the fact that patients with IMGs possess tumors that evolve over the course of years, they often undergo multiple resections and receive several lines of therapy at each progression event. This provides ample opportunity for tumor sampling and histologic immune profiling, which must be employed in future clinical studies to understand cellular determinants of response and recurrence. With the advent of advanced immune profiling techniques, such as single-cell RNA sequencing and multiplexed spatial imaging and transcriptomics, future studies may allow for a more detailed understanding of tumor–immune interactions and guide the timing and combination of immunotherapies.

A critical need is also immune-based biomarkers for patient stratification. While multiple molecular biomarkers (e.g., TP53 mutation, ATRX loss) can aid in the diagnosis and prognostication of IMG, no major biomarkers to date have been validated to reflect immune status. One promising modality is the use of pretreatment TIL infiltration, which may correlate with antigen recognition and might be modulated by subsequent therapies [99]. Though this has been deployed in other solid tumors, a key limitation is that phenotyping of TILs will be necessary to discern if they are ameliorative (e.g., CTL, T_H_1) rather than suppressive (e.g., Treg, T_H_2 subset), which may add complexity to the biomarker interpretation and cost [100]. Relatedly, HLA status is another biomarker with potential relevance to IMG immunotherapy, as it may influence the quantity and quality of targetable tumor neoantigens across patients. Few studies have comprehensively analyzed HLA type across a wide number of IMG cases, with most investigations focused on HLA supertype expression or potential presentation of viral antigens [101,102]. Particularly as IDH1-R132H-specific vaccines progress, comprehensively defining allele-level HLA diversity in IMG will be key to identifying potential determinants of immune recognition.

In addition to tissue analysis, a promising approach for evaluating tumor progression and treatment response in IMGs is liquid biopsy of the cerebrospinal fluid (CSF). This has successfully detected tumor-specific mutations such as IDH1/2, the TERT promoter, and histone H3 in CSF-derived cell-free DNA in gliomas with varying levels of utility [103]. Repeated sampling of the CSF has also been shown to detect long-term variations in allele frequencies which may correlate with treatment response or recurrence [103]. Importantly, liquid biopsy in IMG may eventually allow for instantaneous assessment of a tumor’s molecular status without the need for repeated surgery. This may be useful in better differentiating disease progression from pseudoprogression and recognizing treatment resistance far before clinical recurrence is identified. Finally, integration of liquid biopsy data with known immunologic characteristics of IMG will further enhance patient stratification for immunotherapy clinical trials and investigation of treatment-related changes to the TME.

#### 2.3.4. Prioritization of Experimental Immunotherapies in IMG

Due to its lower incidence relative to GBM, as well as the long periods of intermittent stability exhibited in many cases, designing impactful clinical trials in IMG can be challenging. It is thus important to prioritize therapies that hold the most investigational promise and potential patient benefit for large-scale testing. While multiple therapies hold biological promise, a framework for potentially prioritizing immunotherapies is as follows: (1) Does the therapy augment preexisting immunity, or does it provide new modes of tumor cell engagement? (2) Does the therapy and/or its induced effectors reliably localize to the site of disease within the brain parenchyma? (3) Does the therapy synergize with existing treatment modalities (e.g., chemoradiotherapy or surgery), or should it be investigated in combination with another immunotherapy for maximal potential response? (4) Will the therapy be hampered by the unique immunomodulatory environment produced by 2-HG? (5) Can patients with all grades and histologies of IMG potentially benefit from the proposed therapy, or should it be deployed in a select entity or at a select timepoint in disease history? Aligned with such a schema, we hope multiple future clinical studies may be deployed to rigorously test the clinical activity of immunotherapies within IMG.

## 3. Conclusions

Despite their indolent course, the unique molecular and immunological landscape of IMGs has built a compelling case for novel immunotherapies. Though few candidates have shown definitive clinical efficacy to date, many intriguing immune responses and biomarkers have been identified from approaches such as tumor vaccines and ICIs, and a growing body of preclinical data supports the potential for CAR T cells, myeloid redirection strategies, and OV-based approaches. The next phase of immunotherapy for IMGs will require innovative approaches to optimally mitigate soluble immunosuppressive factors in the TME, optimize in situ T cell persistence, and harness novel antigen targets. We anticipate the outcomes of ongoing clinical trials focused specifically on IMG and look forward to future research that may advance the state of therapeutics for this presently incurable group of tumors.

## Figures and Tables

**Table 1:** Unique clinical, biological, and immunological characteristics of IDH-mutant glioma relative to IDH-wildtype glioblastoma (GBM).

Characteristic	IDH-Mutant Glioma	GBM (IDH-Wildtype)
Prognosis	Better median overall survival (ranges from 21 months to over a decade by grade and entity)	Worse median overall survival (roughly 11 to 15 months)
Tumor growth	Slower growing	More aggressive and faster growing
Metabolic profile	Produces 2-hydroxyglutarate Altered tryptophan metabolism leading to immunosuppression Decreased glycolysis, increased glutaminolysis	Increased glycolysis
Epigenetic profile	Global DNA hypermethylation	Less extensive DNA methylation
Tumor mutational burden	Generally low	Generally low
Known tumor antigens	IDH1-R132H (shared neoantigen); potential TAAs include EGFRvIII, PDGFRA	Many TAAs including EGFR, EGFRvIII, HER2, B7-H3, PTPRZ1, IL-13Rα2
Immune cell infiltration	Lower overall immune cell infiltration	Higher overall immune cell infiltration
CD8+ T cells	Reduced CD8+ T cell accumulation and 2-HG-mediated suppression of effector function	Higher CD8+ T cell infiltration
Regulatory T cells	Fewer regulatory T cells	More regulatory T cells
Myeloid cells	Fewer myeloid cells overall, with grade-dependent increase	Increased myeloid cells, including M2 TAMs and MDSCs
NK cell evasion	Downregulation of NKG2D ligands (ULBP1 and ULBP3)	Relatively higher expression of NKG2D ligands
VEGF expression	Specific increased VEGF expression in higher-grade tumors	High VEGF expression globally

**Table 2:** Overview of ICI clinical trials in recurrent IDH-mutant glioma patients.

NCT No.	Institution	Phase	Treatment Regimen	Status
NCT03576612	Johns Hopkins	I	GMCI + Nivolumab (anti-PD-1) + Radiation Therapy + TMZ	Completed
NCT03991832	University Health Network, Toronto	II	Durvalumab (anti-PD-L1) + Olaparib (PARPi)	Recruiting
NCT03925246	Hôpitaux de Paris	II	Nivolumab	Completed
NCT04056910	University of Pittsburgh Medical Center	II	Nivolumab + Ivosidenib	Completed
NCT02968940	NYU Langone Health	II	Avelumab (anti-PD-L1) + HFRT	Completed
NCT03718767	National Institutes of Health Clinical Center	II	Nivolumab	Recruiting
NCT03557359	Columbia University Medical Center	II	Nivolumab	Active, not recruiting
NCT04160494	Duke University	I	Atezolizumab (anti-PD-L1) + D2C7-IT	Active, not recruiting
NCT03684811	Novo Nordisk A/S	I/II	Nivolumab + FT-2102 + Azacitidine + Gemcitabine + Cisplatin	Completed
NCT03893903	German Cancer Research Center	I	IDH1-R132H-specific vaccine + Avelumab	Active, not recruiting
NCT05484622	Institut de Recherches Internationales Servier	I	Vorasidenib + Pembrolizumab	Recruiting
